# Gut Homing CD4+ and CD8+ T-Cell Frequencies in HIV Infected Individuals on Antiretroviral Treatment

**DOI:** 10.1371/journal.pone.0166496

**Published:** 2016-11-29

**Authors:** Olivia Briceño, Sandra Pinto-Cardoso, Nataly Rodríguez-Bernabe, Akio Murakami-Ogasawara, Gustavo Reyes-Terán

**Affiliations:** Departamento de Investigación en Enfermedades Infecciosas, Instituto Nacional de Enfermedades Respiratorias, Ciudad de México, México; Harvard Medical School, UNITED STATES

## Abstract

The depletion of mucosal CD4+ T-cells occurs early in HIV infection and despite years on antiretroviral treatment (ART), this population never reconstitutes to pre-HIV infection levels. In an effort to understand the effect of ART initiation and different ART regimens on the reconstitution of mucosal T cells within the gut associated lymphoid tissue (GALT), we quantified the frequency of CD4+ and CD8+ T cells expressing the gut homing receptors CCR9 and β7 in peripheral blood (PB) of HIV infected individuals naive to ART and treated individuals on both short-term (less than a year) and long-term ART (more than 2 years). We found that the gut homing CD4+ T cells were depleted in ART-naive individuals and increased after ART initiation but levels were not comparable to HIV uninfected individuals. Gut homing CD4+ T cell activation decreased after ART initiation whilst gut homing CD8+ T cell activation remained elevated in ART experienced individuals, especially in those individuals taking protease inhibitors. Our findings provide new insights into the effects of ART initiation and ART regimens on the frequency and immune status of gut homing CD4+ and CD8+ T cells.

## Introduction

During acute human immunodeficiency virus (HIV) infection, CD4+ T-cells within the gut associated lymphoid tissue (GALT) are depleted [1,2], preferentially the TH17 subset, which plays a key role in the homeostatic maintenance of the intestinal epithelial barrier [3]. The damage suffered by this latter leads to the translocation of bacterial products to the peripheral circulation [4]. Bacterial translocation is one of the main mechanisms responsible for the persistence of chronic immune activation and residual inflammation [5,6]. Indeed, chronic immune activation remains a hallmark of HIV disease progression [7,8] despite successful antiretroviral treatment (ART). Full reconstitution of both peripheral blood (PB) and mucosal CD4+ T-cells is a desirable goal upon ART initiation. However and despite several years on ART, mucosal CD4+ T-cell restoration is fairly modest [9,10]. The factors associated with poor mucosal CD4 T-cells reconstitution are still poorly understood. In 2012, Mavigner and colleagues showed that gut homing CD4+ T-cells remained in circulation due to the lack of proper signaling by the CCR9 ligand, CCL25 [11]. Also, gut CD4+ T-cells, in particular the TH17 population, are susceptible to be infected and killed by HIV [12,13] as the integrin α4β7, necessary for gut homing trafficking, can be used by the virus to infect these cells, further adding to their depletion [14,15]. This in turn has been shown to contribute to the lack of epithelial barrier damage repair [3]. Studying gut homing T-cell frequencies (defined by the expression of α4+β7+ and CCR9+) in PB has been used by many as a useful surrogate to quantify immune reconstitution in the GALT in the absence of gut biopsies [11,16]. In the present study we report the frequency of gut homing CD4+ and CD8+ T-cells, gut homing CD4+ and CD8+ T-cell activation and gut homing TH17 frequencies (expression of the lineage marker CD161+) in PB of HIV-infected individuals naive to ART, ART experienced individuals on short- and long-term treatment, HIV controllers and compared them to HIV uninfected individuals.

## Material and Methods

### Cohort Characteristics

This study was evaluated and approved by the comité de ética en investigación y comité de ética of the Instituto Nacional de Enfermedades Respiratorias (INER) and conducted according to the principles expressed in the Declaration of Helsinki. All participants were adults (over 18 years old) and gave written informed consent. Our cohort was composed of Mexican Mestizo individuals. A total of 76 individuals were recruited for this study (Table 1) and included 5 HIV-uninfected individuals (who tested negative for HIV ELISA test), 6 HIV controllers, defined as HIV infected (HIV+) individuals with plasma Viral Load (pVL) levels <2,000 RNA copies/mL for at least 1 year in absence of ART, 18 HIV+ naive to ART and 47 HIV+ ART-experienced individuals. Our ART-experienced cohort was divided in two groups based on the length of treatment: short (less than a year on ART) and long term (more than 2 years on ART with undetectable pVL).

**Table 1:** Clinical characteristics. of the cohort.

Groups (n = 76)	Gender M/F	Age years	pVL HIV-1 RNA copies/mL	CD4 T Cells/mm^3^	ART regimens	Time on ART
ART-naive (n = 18)	17/1	29 ± 5.9	129,814 ± 540,900	425 ±201	NA	NA
Short-term ART (n = 15)	15/0	31 ± 9.38	<40	302.04 ± 213	EFV/TDC/FTC	Less than 1 year
					EFV/TDC/FTC (n = 15)	
					LPV_r/TDC/FTC (n = 7)	
Long-term ART (n = 32)	29/3	40 ± 8.58	<40	514 ± 267.67	ATV_r/TDF/FTC (n = 8)	9 years ± 2.89
					AZT/3TC/LPV_r (n = 1)	
					TDF/FTC/LPV_r/AZT (n = 1)	
HIV Controllers (n = 6)	3/3	37 ± 15.43	75 ± 175	854 ± 304.93		NA
HIV SN (n = 5)	4/1	41 ±12.98	NA	1,050 ± 205.16		NA

Data is given as median ± standard deviation. Abbreviations: n = number of individuals per group, ART = antiretroviral treatment, M/F = male/female, NA = not applicable, pVL = plasma Viral Load, EFV = efavirenz, TDF = tenofovir, FTC = emtricitabine, LPV = lopinavir, r = ritonavir-boosted, ATV = atazanavir, AZT = zidovudine and 3TC = lamivudine.

### Plasma viral load and CD4 measurements

HIV viral load was determined by automated real time polymerase chain reaction (PCR) using the m2000 system (Abbott, Abbott Park, IL). CD4+ T-cell counts were obtained by flow cytometry using the Trucount Kit in FACSCanto II instruments (BD Biosciences, San Jose, CA).

### Peripheral blood mononuclear cells (PBMCs) isolation

Thirty-six ml of PB were collected from each participant at study entry. PBMCs were isolated by density gradient centrifugation using lymphoprep (Axis-Shield, Oslo, Norway) according to the manufacturer´s instructions. Isolated PBMCs were frozen for batch analysis in 90% fetal calf-serum (Bio-west, California, USA) and 10% dimethyl sulfoxide (DMSO, Sigma-Aldrich, California, USA) and stored in liquid nitrogen until used.

### Immunophenotyping of gut homing CD4+ and CD8+ T cells

**Fig 1:**
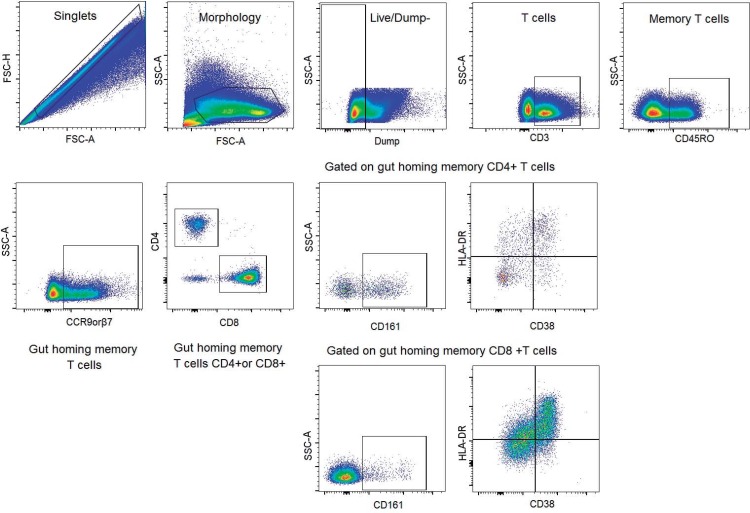
Gating strategy. The gating strategy used for all the samples was to first define singlet and morphology by using forward versus side scatter, followed by the exclusion of dead cells (aqua dye negative events) and the exclusion of CD14, CD19, CD56, CD11c and CD123 positive cells (dump gate). Live cells were gated on CD3+ and CD45RO+ cells. Next, CCR9+ and/or β7+ cells were gated and defined as gut homing T-cells. Then CD4+ and CD8+ T cells were gated and finally the expression of CD161 on the gut homing CD4+ (top) and CD8+ (bottom) T-cells was gated. T cell activation was determined by the simultaneous expression of CD38 and HLADR on gut homing CD4+ and CD8+ T cells.

A combination of monoclonal antibodies were used: anti-CD3 brilliant™ violet (BV) 570 (clone: UCHT1, diluted 1/100, Biolegend, California, USA), anti-CD4 phycoerythrin Cyanine5.5-conjugated (PE-Cy5.5) (clone: MHCD0418, diluted 0.35/100, Invitrogen, USA), anti-CD8 BV605 (clone: RPA-T8, diluted 1/100, Biolegend, California, USA), anti-CD45RO BV650 (clone: UCHL1, diluted 1/50, Biolegend, California, USA), anti-Integrin-β7 phycoerythrin (PE) (clone: FIB27, diluted 1/50, Biolegend, California, USA), anti-CCR9 PE (clone: 1125O9, diluted 3.5/50, BD, California, USA), anti-CD38 BV711 (clone: HIT2, diluted 1/100, Biolegend, California, USA), anti-HLADR BV785 (clone: UCHT1, diluted 1/50, Biolegend, California, USA) and anti-CD161 allophycocyanin (APC) (clone: HP-3G10, diluted 2.5/100, Biolegend, California, USA). Live/Dead Aqua dye (diluted 0.5/100, Invitrogen–Life Technologies, California, USA) was used to exclude dead cells. A dump gate was used to exclude unwanted populations and consisted of anti-CD14 (diluted 0.35/100), anti-CD19 (diluted 0.35/100), anti-CD56 (diluted 0.35/100), anti-CD11c (diluted 0.35/100) and anti-CD123 (diluted 0.45/100), all on BV510 channel (Biolegend, California, USA). As circulating gut homing T-cells compose less than 2% of total PB T-cells [17], we decided to detect both CCR9 and β7 markers in the same channel (PE) to enrich for this gut homing population. After thawing, total PBMCs were washed twice with phosphate-buffered saline (PBS) and counted manually. A total of 5 million PBMCs were stained extracellularly by incubating the cells with the antibody cocktail at laboratory temperature for 15 min. Cells were then washed twice with PBS and fixed in 300ul of 1% paraformaldehyde (Sigma-Aldrich, St. Louis, MO, USA). Samples were acquired on a LSRII cytometer (BD, Biosciences, San Jose California, USA) immediately after staining and fixing. FMO (fluorescence minus one) stained tubes were used as gating controls. Data were collected from single time points. The gating strategy is shown in Fig 1. Quality control was performed in each experiment using BD cytometer Setup & Tracking Beads (BD biosciences, San Jose California, USA). Also, a compensation matrix was calculated and applied individually to each experiment using BD Comp Beads Anti- Mouse Ig, k (BD biosciences, San Jose California, USA). Data was analyzed using FlowJo V10 software (Tree Star Inc.).

### Statistical analysis

Correlations between T cell immunophenotyping and clinical data (Viral load, total CD4 or CD8 T-cell counts and time on ART) were evaluated using spearman's rank correlation coefficient. Clinical data for each individual was obtained on the same day PBMCs were isolated for T cell immunophenotyping. Comparison between groups was done using U-Mann Whitney test and Kruskal-Wallis test correcting for multiple corrections with Dunnett´s post-test. Corrected P values below 0.05 were considered significant. Statistical analyses were performed using GraphPad Prism version 5 (Statcon, La Jolla, CA, USA).

## Results

### The frequency of PB gut homing CD4+ and CD8+ T-cells is altered in HIV infection

**Fig 2:**
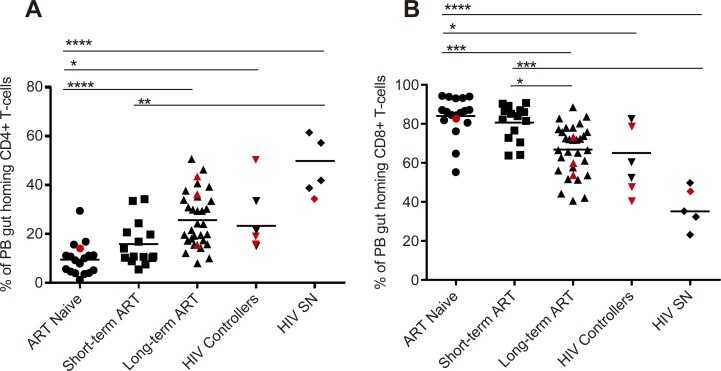
The frequency of PB gut homing CD4+ and CD8+ T-cells is altered in HIV infection. Frequencies (expressed as percentages) of peripheral blood (PB) memory gut homing (CD45RO+ CCR9+ β7+) CD4+ (A) and CD8+ (B) T-cells was determined by flow cytometry on PBMCs of HIV-infected individuals (HIV+), antiretroviral (ART) naïve (n = 18), ART experienced individuals on short-term (n = 15) and long-term treatment (n = 32), HIV controllers (n = 6) and HIV seronegative individuals (SN, n = 5). Scatter plots were used to represent the data. Horizontal lines indicate median values. Each symbol represents one individual. The red symbols represent the females in each group. Groups were compared using Kruskal-Wallis test correcting for multiple comparisons using the Dunnett´s post-test. Only significant corrected p values are shown ***p<0.0005, **p<0.005, *p<0.05. Gender was color-coded as follows: red dots, women and black dots, men.

First, we wanted to determine the frequency of PB gut-homing CD4+ and CD8+ T-cells in our study groups. As shown in Fig 2A, ART-naive individuals had the lowest frequency of PB gut homing CD4+ T-cells compared to individuals on long term ART (p<0.0001), HIV controllers (p = 0.0355) and HIV SN individuals (p<0.0001). Individuals on short-term ART also had lower frequency of PB gut homing CD4+ T-cells compared to HIV SN individuals (p = 0.0036) but not to HIV controllers (p = 0.9615) and long-term ART experienced individuals (p = 0.0659). In contrast, as shown in Fig 2B, HIV+ individuals naive to ART and short-term ART experienced individuals had the highest frequencies of PB gut homing CD8+ T-cells compared to long-term ART individuals (p = 0.0002 and p = 0.0186 respectively), HIV controllers (p = 0.0125 only for ART naive individuals) and HIV SN individuals (p<0.0001 and p = 0.0005 respectively). Frequencies of PB gut homing CD4+ and CD8+ T cells were similar between ART naive and short-term individuals (p>0.99 and p>0.99 respectively, Fig 2A and 2B). Long-term ART experienced individuals and HIV controllers also had similar frequencies of PB gut homing CD4+ and CD8+ T-cells (p>0.99 and p>0.99 respectively; Fig 2A and 2B). No correlations were found between the frequency of PB gut homing CD4+ or CD8+ T-cells and clinical data (total CD4 or CD8 T-cell counts, pVL or time on ART).

### ART-naive and short-term ART experienced individuals have decreased frequency of CD161-expressing gut homing CD8+ T-cells

**Fig 3:**
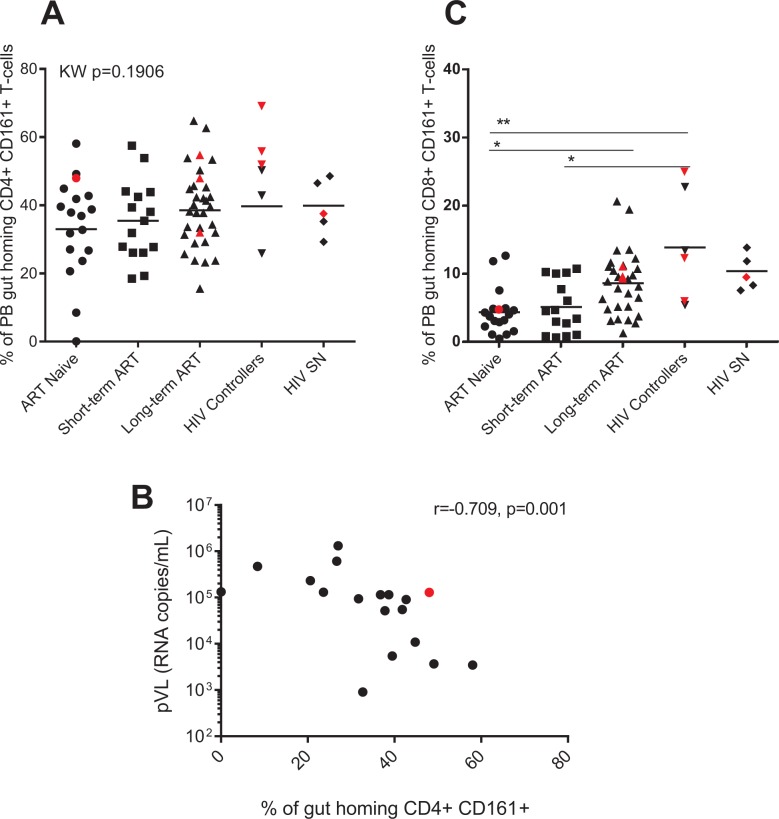
ART-naive and short-term ART experienced individuals have decreased frequency of CD161-expressing gut homing CD8+ T-cells. Frequencies (expressed as percentages) of peripheral blood (PB) memory gut homing (CD45RO+ CCR9+ B7+) CD4+ (A) and CD8+ (C) T-cells expressing CD161 were determined by flow cytometry on PBMCs of HIV-infected individuals (HIV+), antiretroviral (ART) naïve (n = 18), ART experienced individuals on short-term (n = 15) and long-term treatment (n = 32), HIV controllers (n = 6) and HIV seronegative individuals (SN, n = 5). Scatter plots were used to represent the data. Horizontal lines indicate median values. Each symbol represents one individual. The red symbols represent the females in each group. Groups were compared using Kruskal-Wallis test correcting for multiple comparisons using the Dunnett´s post-test. Only significant corrected p values are shown ***p<0.0005, **p<0.005, *p<0.05. Spearman's rank correlation between PB gut homing CD4+ CD161+ T-cells and plasma viral load (pVL) in the ART naive group (B). Gender was color-coded as follows: red dots, women and black dots, men.

Because TH17 cells are essential for maintaining gut mucosa homeostasis, we next assessed the frequency of gut homing CD4+ expressing CD161 (marker of TH17 lineage [18]) in PB. Interestingly, we found no difference in the frequency of gut homing Th17 cells across our study groups (Kruskal-Wallis p = 0.1906, Fig 3A). We did however find a negative correlation between the frequency of PB gut homing CD4+ CD161+ T-cells and pVL in the ART naive group (r = -0.7089; p = 0.001, Fig 3B).

Recently, the expression of CD161+ on CD8+ T-cells has defined a new subset of TH17-like cells (T_C_17) capable of expressing TH17 cytokines (IL-17) [19]. These T_C_17 cells are enriched at sites of inflammation and have been postulated to play a role in disease pathogenesis [20,21]. Therefore, we assessed the frequency of CD161+ expressing gut homing CD8+ T cells in our study groups. As shown in Fig 3C we found that ART naive individuals and short term treated individuals have comparable frequencies of gut homing CD8+ CD161+ T-cells (p>0.99). ART naive individuals showed decreased frequencies compared to long-term ART experienced individuals (p = 0.0115) and HIV controllers (p = 0.0071). The frequency of PB gut homing CD8+ CD161+ T-cells were comparable between long-term ART experienced individuals, HIV controllers and HIV SN individuals (p>0.05, Fig 3C).

### PB gut homing CD4+ T activation decreases with ART, but PB gut homing CD8+ T-cell activation remains elevated in ART experienced individuals on protease inhibitors based therapy

**Fig 4:**
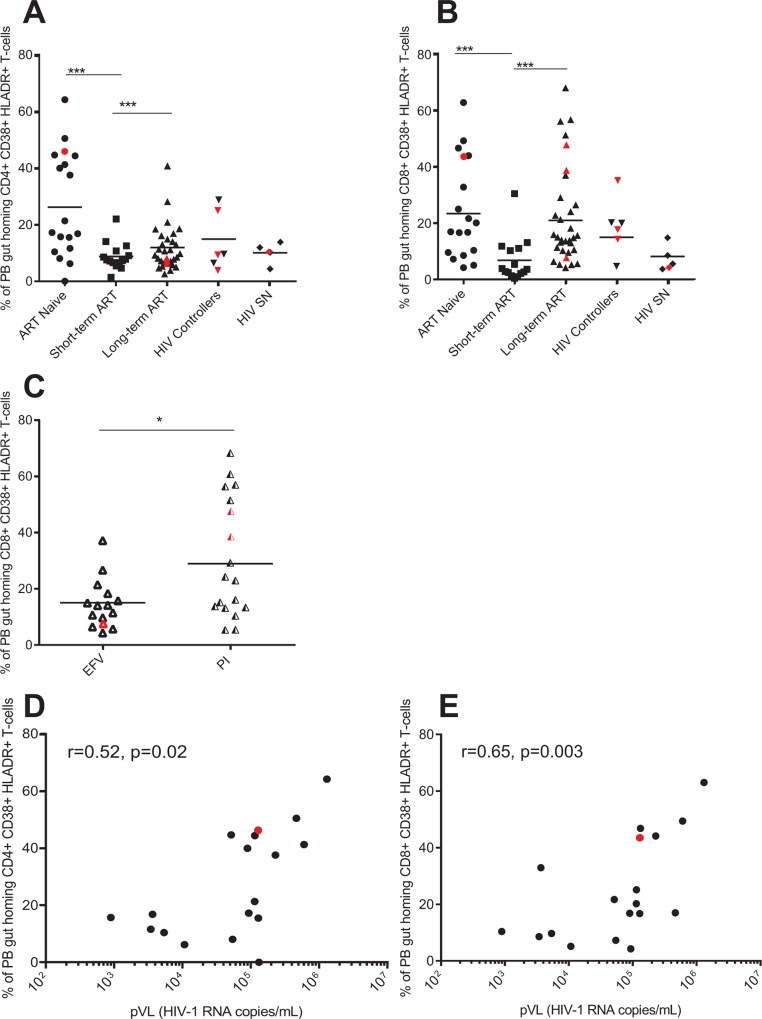
PB gut-homing CD8+ T activation remains elevated in long-term ART experienced individuals on protease inhibitors. Frequencies (expressed as percentages) of peripheral blood (PB) memory gut homing (CD45RO+ CCR9+ B7+) CD4+ (A) and CD8+ (B) T-cells expressing both CD38 and HLADR was determined by flow cytometry on PBMCs of HIV-infected individuals (HIV+), antiretroviral (ART) naïve (n = 18), ART experienced individuals on short-term (n = 15) and long-term treatment (n = 32), HIV controllers (n = 6) and HIV seronegative individuals (SN, n = 5). Frequencies of PB memory gut homing CD8+ T-cells expressing the activation markers CD38 and HLADR in efavirenz (EFV) or protease inhibitors (PI) based therapy is shown in (C). Scatter plots were used to represent the data. Horizontal lines indicate median values. Each symbol represents one individual. The red symbols represent the females in each group. Groups were compared using Kruskal-Wallis test correcting for multiple comparisons using the Dunnett´s post-test. Only significant corrected p values are shown ***p<0.0005, **p<0.005, *p<0.05. Spearman's rank correlation between PB memory gut homing CD4+ (D) and CD8+ (E) T cell activation and plasma viral load (pVL) in the ART naive group. Gender was color-coded as follows: red dots, women and black dots, men.

We then examined the level of T cell activation (via the expression of the classical activation markers CD38+ and HLADR+) of gut homing CD4+ and CD8+ T-cells in PB. As shown in Fig 4A, gut homing CD4+ T cell activation was significantly higher in ART naive individuals compared to short-term ART experienced individuals (p = 0.0008). HIV+ individuals on short and long term ART and HIV controllers reach levels of CD4+ T-cell immune activation comparable to that of HIV SN individuals (p = 0.1897, p = 0.2707 and p = 0.6661 respectively; Fig 4A). However, when assessing gut homing CD8+ T cell activation, we found that ART naive individuals had significantly higher gut homing CD8 T cell activation compared to HIV+ on short-term ART (p = 0.0008, Fig 4B). Interestingly, gut homing CD8+ T cell activation remained elevated in long-term ART experienced individuals when compared to short term treated individuals (p = 0.0006, Fig 4B), indicating that short-term ART has a transient effect in diminishing gut homing CD8+ T cell activation. Because our long-term ART experienced individuals were on two distinct ART regimens based on Efavirenz (EFV) or protease inhibitors (PI, see Table 1), we subdivided the long-term ART experienced cohort into ART regimens to understand the effect of individual ART regimens on gut homing CD8+ T-cells T cell activation. We found that ART experienced individuals on PI had significantly higher gut homing CD8+ T cell activation compared to those on EFV (p = 0.0328) (Fig 4C). Additionally, we found a positive correlation between PB gut homing CD4+ T and CD8+ T cell activation and pVL in the ART naive group (r = 0.51, p = 0.02; Fig 4D and r = 0.65, p = 0.003; Fig 4E respectively), indicating that active viral replication is responsible to gut homing T cell activation in HIV infection.

## Discussion

As far as we are aware, this is the first paper that describes the frequency of CD8+ T cells with gut homing properties in HIV infection. As previously shown, CD8+ T cells are essential for controlling viral replication [22,23]. We found an accumulation of PB gut homing CD8+ T cells in ART naive individuals as compared to HIV SN individuals. Whether this result can be explained by an attempt from the immune system to control HIV replication in the GALT by recruiting more CD8+ T cells with homing properties or a lack of proper signaling to the gut as described for CD4+ T cells [11] remains to be elucidated. Further research is needed to determine the role of gut homing CD8+ population in HIV infection. Interestingly, cytotoxic CD8+ T-cells with gut homing properties expressing the TH17 lineage marker CD161 have been recently described [19,20]. These CD161 +CD8+ T cells are polyfunctional [21] and have been shown to control viral infections [24]. They have also been shown to negatively correlated with CD8+ T cell immune activation in HIV infected individuals [25]. In our study, we found that PB gut homing CD161+ CD8+ frequency were depleted both ART naive and short-term ART experienced. Long-term ART experienced individuals and HIV controllers seemed to recover this population, as frequencies were comparable to HIV SN individuals. It is possible that this polyfunctional population could be associated with the functional immune responses that have been previously reported for HIV-controllers [26,27]. Tracking gut homing CD161+ CD8+ T-cells in PB could be used as a biomarker of effective immune responses reconstitution in the gut during HIV infection.

In this study, we showed that the frequency of gut homing CD4+ T-cells is significantly reduced in HIV infected individuals, in particular, in ART naive individuals. These results are in agreement with previous publications [28] but in disagreements with others [11]. The discrepancy in the results could be explained by differences in the clinical characteristics of the cohorts and/or the markers used to identify gut homing populations. It is well known that the TH17 subset arises from T-cells expressing the lineage marker CD161 and plays a fundamental role on the homeostatic maintenance of the GALT [18,29]. Contrary to what was expected, we found no differences in the frequencies of gut homing CD4+ CD161+ in our study groups. Whether this gut homing CD4+ CD161+ population found in PB is actually representative of Th17 frequencies in the GALT and whether they are responsible for maintaining gut Th17 population at levels comparable to an HIV uninfected individual remains to be elucidated. Also, we did find a negative correlation between the frequencies of gut homing CD4+ CD161+ with the pVL in the ART naïve group possibly indicating that active viral replication could be depleting this population.

We could not find previous reports that studied the effect of different ART regimens on gut homing CD4+ and CD8+ T cell activation. As expected, levels of CD4+ T immune activation decreased to levels comparable to HIV SN individuals. But when looking at gut homing CD8+ T cell activation, we found a transient decrease in PB gut homing CD8+ T cell activation upon ART initiation. Moreover, we found a positive correlation between the pVL with the frequency of activated gut homing CD4+ and CD8+ T-cells, as previously reported [28,30]. Interestingly, when looking at ART regimens separately, we found that ART experienced individuals on PI had significantly higher gut homing CD8+ T cell activation that those on EFV. Our data suggest that activated CD8+ T-cells are preferentially recruitment to the gut in those individuals on PI-based therapy, possibly as a response to more gut damage. Further research is needed to address the effect of different ART regimens on the reconstitution of the GALT.

We are aware that our study has several limitations. First the number of individuals in our HIV SN and short-term ART experienced group are small. Also, the results are based on a descriptive cross-sectional cohort study performed in peripheral blood. Further studies, including the use gut biopsies, are needed to confirm these results.

To conclude, our study sheds some lights into the effect of ART initiation and ART regimens on the frequency and activation status of gut homing CD4+ and CD8+ T-cells in HIV infection.
